# Methanotroph biotechnology: An annotated selection of World Wide Web sites relevant to the topics in microbial biotechnology

**DOI:** 10.1111/1751-7915.12101

**Published:** 2013-12-10

**Authors:** Lawrence P Wackett

**Affiliations:** Department of Biochemistry, Molecular Biology and Biophysics, BioTechnology Institute, University of MinnesotaSt Paul, MN, 55108, USA

## Methane to liquid fuel

http://ens-newswire.com/2013/01/04/biotech-bacteria-could-convert-methane-to-liquid-diesel/

With the recent drop in natural gas prices in the United States, there is heightened interest to use methanotrophic bacteria to produce commercial products such as liquid fuels. This project was recently funded to explore this paradigm.

## Methane-oxidizing bacteria for biotechnology

http://onlinelibrary.wiley.com/doi/10.1002/elsc.200900093/abstract

This paper describes a bacterium that grows on methane and produces copious amounts of polyhydroxy butyrate.

## Methane-to-liquid fuels workshop

http://arpa-e.energy.gov/?q=arpa-e-events/biological-technologies-methane-liquid-fuels-workshop

This page contains notes from a workshop at which approaches were discussed for converting methane to liquid biofuels, largely by biological mechanisms.

## Methanotrophic microbiomes

https://biblio.ugent.be/publication/3166868

This page links to a dissertation focused on studies of methanogenic communities with the goal of mitigating against atmospheric methane release.

## Methylococcus capsulatus

http://microbewiki.kenyon.edu/index.php/Methylococcus_capsulatus

*Methylococcus capsulatus* is a methanotrophic bacterium that has been well studied and is one of two major experimental model systems for understanding the biochemistry of methanotrophy.

## *Methylosinus trichosporium* OB3b: BioCyc

http://biocyc.org/MOB3B/organism-summary?object=MOB3B

*Methylosinus trichosporium* is a methanotrophic bacterium that has been well studied and is one of two major experimental model systems for understanding the biochemistry of methanotrophy. This BioCyc page contains metabolic reconstruction information derived from the genome-sequencing project for this organism.

## Calysta

http://www.calystaenergy.com/index.php

Calysta is a commercial entity that has the goal of converting methane to fuels and chemicals via biological processes using methanotrophic bacteria.

## Advanced Research Projects Agency-Energy gas to fuels projects

http://www.greencarcongress.com/2013/09/20130919-arpae.html

This page contains a list and a description of projects funded by ARPA-E that are focused on converting natural gas to liquid fuels.

## Non-methanotroph in methanotroph environment

http://www.ncbi.nlm.nih.gov/pubmed/23765894

This paper shows methane oxidation by a non-methanotrophic bacterium containing an alkane monooxygenase enzyme.

## Methanotroph ecology for gas prospecting

http://www.ncbi.nlm.nih.gov/pubmed/20107985

Deoxyribonucleic acid sequencing can be used to identify methanotrophic community signatures that might be indicative of natural gas being below the surface in a given region.

## *Methylmicrobium buryatense* 5G genome

http://genomea.asm.org/content/1/4/e00053-13.full

*Methylmicrobium buryatense* 5G is a fast-growing methanotrophic bacterium, and thus it may have commercial value; this study describes its genome sequence.

## Methane monooxygenase: Wikipedia

http://en.wikipedia.org/wiki/Methane_monooxygenase

This general article describes properties of both the soluble and particulate methane monooxygenases.

## Particulate methane monooxygenase X-ray structure

http://www.rcsb.org/pdb/explore.do?structureId=1YEW

This Protein Data Bank (PDB) page has links to structural information for the membrane-bound copper-containing methane monooxygenase found within methanotrophic bacteria.

## Soluble methane monooxygenase structures

http://www.rcsb.org/pdb/results/results.do?qrid=80EA0197&tabtoshow=Current

This PDB page has links to structural information for the cytoplasmic iron-containing methane monooxygenase found within methanotrophic bacteria.

## BRENDA: particulate methane monooxygenase

http://www.brenda-enzymes.org/php/result_flat.php4?ecno=1.14.18.3

This BRENDA pages contains extensive information regarding the properties of particulate methane monooxygenase, including its substrate range.

## BRENDA: soluble methane monooxygenase

http://www.brenda-enzymes.org/php/result_flat.php4?ecno=1.14.13.25

This BRENDA pages contains extensive information regarding the properties of the soluble methane monooxygenase, including its broad substrate range.

## Methane biofiltration systems

http://www.ucalgary.ca/mehrotra/files/mehrotra/2011-Hettiarachchi-Hettiaratchi-Mehrotra-Kumar-EnvironmentalPollution.pdf

Environmental methane releases are of growing concern because of increases in oil extraction from oil sands and via hydraulic fracturing. One method of detailing with methane emissions where gas harvesting is not possible is to use methane biofilters.

